# Identification of dysregulated miRNAs and their regulatory signature in glioma patients using the partial least squares method

**DOI:** 10.3892/etm.2014.2041

**Published:** 2014-10-31

**Authors:** JIAJUN SHOU, SHIXIN GU, WENTAO GU

**Affiliations:** Department of Neurosurgery, Huashan Hospital, Fudan University, Shanghai 200040, P.R. China

**Keywords:** glioma, partial least squares, microRNA, target gene, expression profile, survival

## Abstract

Using microarray data, the present study identified differentially expressed microRNAs (miRNAs) and evaluated their regulatory characteristics in high-grade glioma patients, with the aim to further the understanding into the underlying etiology of the condition. Previously, studies have generally implemented regression or variance analysis, which ignores various background biological factors. However, in the present study, analysis was performed with microarray data collected from the Gene Expression Omnibus database using a partial least squares-based method, which is more sensitive in handling microarray data. Among the six identified differentially expressed miRNAs, hsa-miR-21 and hsa-miR-612 have been previously reported to be associated with glioma. In addition, the remaining miRNAs, hsa-miR-4680, hsa-miR-1908, hsa-miR-4656 and hsa-miR-4467, may also contribute to glioma progression since they are all associated with the tumorigenesis of other types of cancer. Moreover, the expression levels of hsa-miR-1908, hsa-miR-4656 and hsa-miR-4680 have been identified to significantly correlate with the survival rate. Enrichment analysis of the dysregulated target genes revealed that the selected miRNAs primarily affect biological processes in the nervous system and the protein phosphorylation process. Therefore, the results may offer a new understanding into the pathogenesis of high-grade glioma.

## Introduction

Gliomas are a type of tumor that arise from glial cells, constituting ~30% of brain and central nervous system tumors and 80% of malignant brain tumors (1). To date, the prognosis of high-grade (stage III–IV) glioma cases remains poor. microRNAs (miRNAs) are small non-coding RNAs, comprised of 18–22 nucleotides, that regulate gene expression and control various biological processes (2) through binding to the 3′-untranslated region of mRNAs. Subsequently, the stability and translation of the target mRNAs are affected. miRNAs have recently been identified as crucial factors in tumorigenesis, but also tumor aggressiveness (3,4). In addition, miRNAs are hypothesized to be widely dysregulated in cancer; thus, may serve as potential markers for cancer diagnosis, prognosis and treatment (5). miRNAs have also been reported to be associated with the pathogenesis and anticancer treatment sensitivity of gliomas (6). Therefore, the identification of differentially expressed miRNAs and the determination of their regulatory role in high-grade glioma patients may help to further the understanding into the underlying etiology.

High-throughput microarray technology facilitates the investigation of characteristics that underlie the progression of cancer. Several studies have investigated the miRNA expression signature in glioma patients (7,8). However, these studies have generally implemented regression or variance analysis, which is unable to evaluate unaccounted array specific factors. Partial least squares (PLS) analysis has been demonstrated to be effective and more sensitive in handling microarray data (9,10). A previous study used this method on other complex diseases and demonstrated the feasibility (11). Thus, identifying the miRNA expression signature of glioma patients using this method may conduce to a new understanding of the disease progression.

In the present study, to identify the differentially expressed miRNAs and determine their regulatory characteristics in high-grade glioma patients, PLS analysis was performed using microarray data collected from the Gene Expression Omnibus (GEO) database. Survival analysis of the selected miRNAs was conducted to analyze the effect of these miRNAs on the prognosis of the patients. In addition, dysregulated miRNAs and target mRNAs were used to construct a regulatory network. Pathway and Gene Ontology (GO) enrichment analysis of the dysregulated target genes was also used to evaluate the biological effects of the differentially expressed miRNAs.

## Materials and methods

### Microarray data

An expression profile data set from the GEO database (GSE4412) was used, which included the transcription profile of 24 grade III and 50 grade IV glioma patients. All the RNA samples were extracted from fresh frozen tumor tissues that had been collected during surgical treatment. The data set was based on two Affymetrix platforms (GPL96 and GPL97).

### Identification of differentially expressed miRNAs

Raw data of all the samples, including CEL and simple omnibus format in text-formatted files, were obtained from the GEO database. Following quality control, normalization of the raw intensity values was conducted using a Robust Multi-array Analysis (RMA) (12) procedure. Neutralization of background noise effects and processing artifacts was performed with model-based background correction, and expression values of all the probes were aligned to a common scale using quantile normalization. The log_2_-transformed RMA values of all the probes were subsequently used in PLS analysis to estimate their effects on the grade III and IV samples. Briefly, PLS latent variables were initially calculated with the non-linear iterative partial least squares algorithm (13); subsequently, the effects of the probe expression values on the disease status were estimated using variable importance in the projection (VIP) (14). Finally, the false discovery rate (FDR) of each probe was calculated based on the empirical distribution of the PLS-based VIP scores, generated by a permutation procedure (n=10,000). miRNAs with a FDR of <0.01 were considered to be significant differentially expressed miRNAs. The aforementioned procedures were performed with R software (version 3.0.0; http://www.r-project.org/), including BioConductor (http://www.bioconductor.org/.), limma packages (3.12.1) and libraries (15).

### Survival analysis

To investigate the contribution of the differentially expressed miRNAs to the survival time following surgery, survival analysis was performed. For each miRNA, the samples were separated into two classes using a K-mean algorithm based on the expression value. Using the survival time or last follow-up time of the patients, the log-rank test was used to investigate whether the two classes were significantly different from each other. P<0.05 was considered to indicate a statistically significant difference; thus, miRNAs with these values were found to be significantly associated with the survival rate.

### Target gene prediction and network construction

Target gene prediction for the differentially expressed miRNAs was performed using currently available methods, including microT (16), miRanda (17), mircode (18) and TargetScan (19). Target genes supported by at least two methods were used in further analysis. Differentially expressed genes were selected using the same procedure as for the detection of differentially expressed miRNAs. Target genes that were identified to be differentially expressed in the grade III and IV samples were subsequently used to construct a regulatory network of selected miRNAs using Cytoscape software (V 2.8.3, http://www.cytoscape.org/) (20).

### Enrichment analysis

To estimate the biological effects of the differentially expressed miRNAs, enrichment analysis was performed for the differentially expressed target genes. Target genes were firstly annotated based on the Kyoto Encyclopedia of Genes and Genomes (KEGG) pathways (http://www.genome.jp/kegg/) and GO database. A hypergeometric distribution test was then used to identify the pathways and GO items enriched with dysregulated miRNA target genes.

## Results

### Identification of differentially expressed miRNAs

As shown in Table I, six miRNAs were identified to be differentially expressed in the grade III and IV glioma patients, including two downregulated miRNAs (hsa-miR-4680 and hsa-miR-1908) and four overexpressed miRNAs (hsa-miR-4656, hsa-miR-4467, hsa-miR-612 and hsa-miR-21).

### Survival analysis

**Figure 1:**
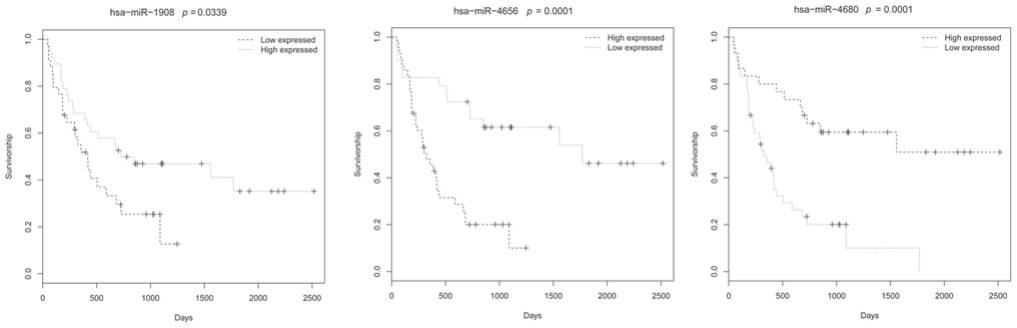
Survival curve of the three miRNAs that were identified to be associated with the survival rate of the patients. miRNAs, microRNAs.

Survival analysis of these miRNAs revealed that three miRNAs, hsa-miR-1908, hsa-miR-4656 and hsa-miR-4680, were significantly associated with the survival rate of the patients, with P-values of 0.0339, 0.0001 and 0.0001, respectively (Fig. 1).

### Target gene prediction and network construction

**Figure 2:**
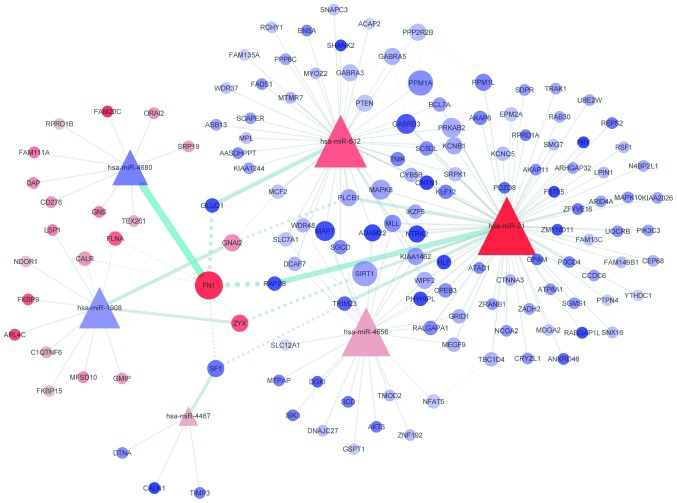
Regulatory network constructed with dysregulated miRNAs and target genes. Interactions between target genes are shown with a dotted line. Items in red represent overexpressed molecules in grade IV patients, while those in blue represent downregulated molecules in grade IV samples. Molecules with a higher number of interactions are shown in a bigger size. miRNAs, microRNAs.

Target gene prediction and PLS analysis identified 139 differentially expressed target genes, and a network was constructed using these miRNAs and target genes (Fig. 2). The degree of each node was defined as the number of its interactions in the network. Nodes with higher degrees are shown as a bigger size. Among the six miRNAs, hsa-miR-21 was found to have a higher number of interactions compared with the other miRNAs. In the three overexpressed miRNAs, hsa-miR-21, hsa-miR-612 and hsa-miR-4656, a number of the target genes were found to be shared. Among the target genes, *FN1* was detected to have the highest degree.

### Enrichment analysis

Enrichment analysis of the differentially expressed target genes identified two KEGG pathways and four GO items over-represented with dysregulated target genes (Table II). The two KEGG pathways were retrograde endocannabinoid signaling and dopaminergic synapse, which are both involved in the nervous system. In addition, one of the GO items, the postsynaptic membrane (GO:0045211), is also associated with the nervous system. The remaining three GO items were shown to be associated with the protein phosphorylation process, including protein serine/threonine phosphatase activity (GO:0004722), JUN phosphorylation (GO:0007258) and protein dephosphorylation (GO:0006470).

## Discussion

To identify differentially expressed miRNAs and determine their regulatory characteristics in high-grade glioma patients, PLS analysis was performed. In total, six miRNAs were identified to be dysregulated. Among them, hsa-miR-21 has been previously reported to exhibit a significant correlation with tumor grade and prognosis (21,22). In addition, the expression of hsa-miR-612 has been reported to be associated with magnetic resonance imaging features of glioblastoma multiforme (23). The results of the present study further confirmed the involvement of the two miRNAs in the progression of glioma. In addition, one of the target genes of miR-612 is PTEN. PTEN is a tumor suppressor that negatively regulates the protein kinase B/Akt-dependent cell survival pathway (24). Therefore, depression of PTEN may impact its negative regulation of tumor cell survival and contribute to the deterioration of the disease. None of the remaining four miRNAs have been previously reported to be associated with glioma. However, hsa-miR-1908 has been reported to be associated with the metastasis or tumorigenesis of other types of tumor, including chordomas (25), hepatoma (26) and melanoma (27). In addition, hsa-miR-4680, hsa-miR-4656 and hsa-miR-4467 have been hypothesized to be associated with breast cancer (28). For miR-4680, one of its target genes is *FN1*, which is a hub gene that was found to have the highest degree among the target genes. The protein encoded by this gene is fibronectin, which is involved in cell adhesion and migration processes. A previous study reported a significant correlation between this gene and malignant glioma (29), indicating the potential regulatory mechanism of miR-4680 in the development of glioma. Survival analysis also revealed that the expression levels of hsa-miR-1908, hsa-miR-4656 and hsa-miR-4680 were significantly associated with the survival rate of the patients (Fig. 1). Moreover, the constructed network of dysregulated miRNAs and target genes revealed that hsa-miR-21, hsa-miR-612 and hsa-miR-4656 share a number of target genes, indicating that they may affect similar biological processes. Thus, further investigation of these miRNAs is warranted.

Pathway enrichment analysis of the dysregulated target genes revealed that the differentially expressed miRNAs primarily affect pathways in the nervous system, including retrograde endocannabinoid signaling and the dopaminergic synapse. GO analysis additionally revealed the over-representation of dysregulated target genes in the protein phosphorylation process. The GO:0004722 item of protein serine/threonine phosphatase activity exhibited the most significant enrichment. A previous study reported the alteration of striatal dopaminergic function in glioma development (30). Furthermore, dysregulation of serine/threonine phosphatase calcineurin has also been reported in grade IV astrocytoma tumor tissue (31). The enrichment analysis results further confirmed the effects of the dysregulated miRNAs.

In summary, using an expression profile from the GEO database, PLS-based analysis was performed to identify differentially expressed miRNAs and evaluate their regulatory characteristics in high-grade glioma patients. In total, six miRNAs were identified to be dysregulated, including three miRNAs significantly associated with the survival rate of the patients. Enrichment analysis of the dysregulated target genes revealed that the differentially expressed miRNAs predominantly affected biological processes associated with the nervous system and the protein phosphorylation process. Thus, the results may offer a new understanding into the pathogenesis of high-grade glioma.

## Figures and Tables

**Table I:** Differentially expressed miRNAs.

miRNAs	Fold change	FDR
hsa-miR-4680	−0.7602	0.0014
hsa-miR-1908	−0.6327	0.0074
hsa-miR-4656	0.4312	0.0127
hsa-miR-4467	0.3912	0.0164
hsa-miR-612	0.9768	0.0220
hsa-miR-21	1.4927	0.0273

FDR, false discovery rate; miRNAs, microRNAs.

**Table II:** Pathways and GO items enriched with differentially expressed genes.

Parameter	Description	Class	P-value
KEGG pathway			
hsa04723	Retrograde endocannabinoid signaling	Nervous system	0.0054
hsa04728	Dopaminergic synapse	Nervous system	0.0308
GO item			
GO:0004722	Protein serine/threonine phosphatase activity	Function	0.0012
GO:0007258	JUN phosphorylation	Process	0.0115
GO:0006470	Protein dephosphorylation	Process	0.0115
GO:0045211	Postsynaptic membrane	Component	0.0391

KEGG, Kyoto Encyclopedia of Genes and Genomes; GO, Gene Ontology.
